# Implementation of a Departmental Female Emergency Medicine Physician Group

**DOI:** 10.5811/westjem.2018.11.39827

**Published:** 2018-12-10

**Authors:** Kendra P. Parekh, Tara Overbeeke, R. Maglin Halsey-Nichols

**Affiliations:** Vanderbilt University Medical Center, Department of Emergency Medicine, Nashville, Tennessee

## Abstract

Gender disparities exist in academic emergency medicine (EM). We developed and implemented a female EM physician group – Women in Academic Emergency Medicine (WAM) – to support female EM residents, fellows, and faculty. The goal of WAM is to provide a support system through mentorship, education, and outreach. A targeted needs assessment was completed to identify goals and objectives specific to our department. In the first full year of implementation, WAM hosted eight events, including three topical dinners and one formal panel. Of 42 female faculty and residents, 40 (95%) attended at least one WAM event, and all (20/20) of the female faculty strongly supported WAM. WAM advocated for increased female physician representation on the department’s Physician Executive Leadership Group and preservation of dedicated lactation space in the emergency department. Using a needs assessment, the process of developing WAM can be replicated in any department to create a female physician group.

## BACKGROUND

Despite an increased number of female physicians in the workforce, gender disparities continue to exist in academic medicine, and the specialty of emergency medicine (EM) is no exception.^1^ Within EM, disparities have been found in regard to salary, career advancement, and resource allocation.^2^–^4^ A proposed method to address these disparities is the promotion of supportive environments with a focus on the organizational context and culture in which women work.^5^ To support female emergency physicians (EP) in our department, we developed and implemented Women in Academic Emergency Medicine (WAM). Although female faculty groups often exist at the national and institutional level, we found no description in the EM literature of the development of a formal female faculty group at the departmental level. Thus, we present a description of our developmental process, implementation, and initial outcomes to serve as a model.

## OBJECTIVES

The goal of WAM is to provide a support system for female physicians through mentorship, education, and outreach. Our objectives are to 1) promote professional advancement and leadership skills of female EPs; 2) provide mentorship opportunities and mutual support; 3) develop and present educational programming pertinent to female EPs; 4) connect members with other female physicians locally and nationally; and 5) engage the greater community through outreach opportunities.

## CURRICULAR DESIGN

We used Kern’s six steps of curriculum development to create the curriculum for WAM, and all female EPs were invited to participate in the development process.^6^ We conducted a general needs assessment via literature review, and completed a targeted needs assessment through an anonymous online survey of female faculty and focused interviews of female residents (Appendix).^7^ Goals and objectives were developed in an iterative fashion over email and subsequently deliberated among female faculty in small group sessions. Based on our targeted needs assessment, the guiding principles in implementation were to empower female physicians, maximize social contact, and leverage available resources.

Two leaders (KP, TO) planned events that covered the three pillars of WAM, and the chair agreed to support the events. Events were planned in advance, and the clinical schedule was reviewed to ascertain dates and times when the fewest female EPs were working in the emergency department (ED). In the first full year of implementation (2016–2017 academic year), WAM hosted eight formal activities, including three topical dinners and one formal seminar (Table).

## IMPACT/EFFECTIVENESS

We used Kirkpatrick’s model to evaluate WAM in the first year of implementation.^8^ Completion of events as scheduled and attendance at events measured reaction to the group. All scheduled events were successfully completed with 95% (40/42) of female faculty and residents attending at least one WAM event, and 90.5% (38/42) attending more than one event. All (20/20) of the female faculty strongly or very strongly supported WAM.

Through informal measures, WAM influenced behavior and demonstrated results. By hosting a virtual meeting with resident alumna, two physicians obtained professional opportunities that were not previously available. From a departmental perspective, WAM increased open conversations about disparities among male and female physicians. WAM gave a voice to female faculty and advocated for increased female physician representation on the department’s Physician Executive Leadership Group and for preservation of dedicated lactation space in the ED. We believe the specific goals and objectives coupled with the formal planning and departmental support of WAM allowed for a broader impact than an ad hoc faculty group.

Lessons learned in the first year of implementation included the following: ensuring inclusivity and not focusing solely on motherhood; engaging faculty in multiple ways; thinking critically about which activities are best for resident inclusion; and empowering female faculty as agents of institutional change. In response to these challenges, we have increased the diversity and timing of events, created time for faculty-only events, appointed a director of wellness, and developed a resident liaison position.

The process of developing WAM can be replicated in any department to develop a female physician group. The detailed needs assessment serves as the cornerstone of successful implementation. WAM has the potential to impact recruitment of female faculty and residents as well as faculty retention, promotion, and wellness. Data collection in these areas is ongoing and further research is needed to explore the full impact of the program on female physicians.

## Supplementary Material



## Figures and Tables

**Table:** Activities in the first year of implementation of Women in Academic Emergency Medicine.

Month	Activity
July	Welcome dinner (topic: time management)
August	Discussion of needs-assessment results
September	Emergency medicine day of service at Project Cure
October	Faculty dinner (topic: sexism in medicine)
December	Mentoring breakfast with grand rounds speaker
December	Holiday cookie exchange
January	Mentoring breakfast with grand rounds speaker
January	Winter wellness week
April	Educational series: Money Matters
May	Graduating resident appreciation dinner
