# Supplementary material for: Implementation of a Departmental Female Emergency Medicine Physician Group
Source: West J Emerg Med. 2018 Dec 10;20(1):98–9. doi: 10.5811/westjem.2018.11.39827 (PMC6324705; doi:10.5811/westjem.2018.11.39827)
Supplement: Supplementary file 1 [file wjem-20-98-s001.docx]

Appendix: Female Faculty Needs Assessment Survey and Resident Interview Questions

Female Faculty Needs Assessment Survey^1^

What is your age?

<30

30-39

40-49

50-59

>60

I do not wish to answer

What is your academic appointment?

Full time, full effort

Full time, partial effort

Part time

I do not wish to answer

How many years have you been in academics?

0-5

6-10

11-15

16-20

>20

I do not wish to answer

Describe your current level of satisfaction with your career:

Not at all satisfied

Somewhat satisfied

Neither satisfied nor dissatisfied Satisfied

Very satisfied

I do not wish to answer

Is professional advancement important to you?

Yes

No

How important professional advancement to you?

Not at all important

Somewhat important

Important

Very important

I do not wish to answer

Do you expect to get promoted and/or move into a leadership position in the next 5 years?

Yes

No

How satisfied are you with the pace of your professional advancement?

Not at all satisfied

Somewhat satisfied

Neither satisfied nor dissatisfied

Satisfied

Very satisfied

I do not wish to answer

Please how likely you are to do the following:

Likelihood of recommending Vanderbilt to prospective female residents

Not likely

Somewhat likely

Likely

Very likely

Not sure

Likelihood of recommending Vanderbilt to prospective female faculty

Not likely

Somewhat likely

Likely

Very likely

Not sure

Likelihood of remaining in academic medicine for the next 5 years

Not likely

Somewhat likely

Likely

Very likely

Not sure

Likelihood of remaining at Vanderbilt for the next 5 years

Not likely

Somewhat likely

Likely

Very likely

Not sure

Rate your agreement with the following statements.

Male and female faculty members have access to the same professional opportunities within the department.

Strongly agree

Agree

Disagree

Strongly disagree

I am not sure

Male and female faculty members in similar positions in the institution are paid comparable salaries.

Strongly agree

Agree

Disagree

Strongly disagree

I am not sure

Institutional promotions are awarded to both male and female faculty members in a similar fashion.

Strongly agree

Agree

Disagree

Strongly disagree

I am not sure

Women do not pursue academic promotion due to lack of departmental support.

Strongly agree

Agree

Disagree

Strongly disagree

I am not sure

Male and female faculty members are offered similar protected time for research activities.

Strongly agree

Agree

Disagree

Strongly disagree

I am not sure

Male and female faculty members are offered similar protected time for teaching activities.

Strongly agree

Agree

Disagree

Strongly disagree

I am not sure

Which faculty development event(s) have you attended? Select all that apply.

Junior faculty development event

VUSM/VUMC faculty development event

National conference

Other

How supportive are you of a female faculty group?

Very supportive

Supportive

Somewhat supportive

Not supportive

I am not sure

What recommendations do you have for implementing a female faculty group?

What type of faculty development activities interest you? Select all that apply.

Coaching

Dinners

Lunch seminars

Mentoring

Panel discussions

Speed networking

Webinars

Other

What specific faculty development topics interest you? Select all that apply.

Appointment and promotions

Coaching

Communication

Conflict management

Gender and diversity

Grant writing

Mentoring

Networking

Pathways to leadership

Resiliency

Strength finding

Time management

Work-life integration/balance

Please describe any other topics that interest you.

Resident Interview Questions

We are considering starting a female EM physician group in the department.

1. What do you think about this?
2. What would you like the group to accomplish or provide?
3. What types of activities would interest you?
4. Adapted from Spalluto LB, Arleo EK, Macura KJ, et al. A Leadership Intervention to Further the Training of Female Faculty (LIFT-OFF) in Radiology. Acad Radiol. 2017;24(6):709-716.
